# Incidence and Predictors of Pulmonary Tuberculosis among Children Who Received Antiretroviral Therapy (ART), Northwest Ethiopia: A Multicenter Historical Cohorts Study 2009–2019

**DOI:** 10.1155/2022/9925693

**Published:** 2022-01-29

**Authors:** Fassikaw Kebede, Habtamu Tarekegn, Mulugeta Molla, Dube Jara, Abebe Abate

**Affiliations:** ^1^Woldia University, College of Health Sciences, School Public Health, Department of Epidemiology & Biostatics, Woldia, Ethiopia; ^2^Pawe Health Science College, Department of Pharmacy, Pawe Metekel, Ethiopia; ^3^Debre Tabor University, College of Medicine and Health Science, Department of Pharmacy, Debre Tabor, Ethiopia; ^4^Debre Markose University, College of Medicine and Health Science, Department of Public Health, Debra Markose, Ethiopia

## Abstract

The human immune deficiency virus (HIV) is the strongest risk factor for endogenous reactivation of pulmonary tuberculosis (PTB) through target reduction of CD4, T-lymphocytes, and cellular immune function. Almost one-third of deaths among people living with HIV are attributed to tuberculosis. Despite this evidence, in Ethiopia, information is scarce and meager regarding PTB incidence after ART initiated for seropositive children. *Methods*. Facility-based multicenter historical cohort was conducted among 721 seropositive children after initiating ART from January 1, 2009, to December 31, 2019. Data from the records of children were extracted using a standardized checklist. The collected data were entered using Epi-Data version 4.2 and exported to STATA (SE) R-14 version statistical soft wares for further analysis. Bivariable and multivariable Cox regression analyses were conducted to identify predictors of PTB incidence. *Results*. Seven hundred twenty-one (*N* = 721) seropositive children were included with a mean (±SD) age of 118.4 ± 38.24 months. During the follow-up periods, 63 (15.2%) participants developed new cases of TB; majority (61/63, 96.8%) of them were PTB. The overall incidence rate and the median (±IQR) time of PTB reported were determined as 5.86 per 100 child years (95% CI: 4.58, 7.5) and 17.8 (±11) months, respectively. At baseline, children being severely stunted (AHR = 2.9 : 95% CI, 1.2–7.8, *P*=0.03), with Hgb ≤10 mg/dl (AHR = 4.0; 95% CI, 2.1–8.1, *P*=0.001), and not given isoniazid and cotrimoxazole preventive therapy (AHR = 2.4; 95% CI: 1.2; 5.1, *P*=0.001) (AHR = 2.5; 95% CI, 1.4–4.7, *P*=0.021) were significantly associated with PTB incidence. *Conclusion*. A high incidence rate of PTB was observed in our study as compared with the previous finding in Ethiopia. Cases at baseline not taking IPT and CPT, being severely stunted, and having low hemoglobin (≤10 mg/dl) levels were found to be at higher risk of developing PTB.

## 1. Introduction

The human immune deficiency virus (HIV) is the strongest risk factor for latent (PTB) or new infection of tuberculosis (TB) through reduction of CD4 T-lymphocytes and cellular immune function [1]. Despite this fact, both (TB/HIV) coinfections are bidirectional and comrade each other, in which HIV sustains the progression of latent tuberculosis bacilli into active TB, while tuberculosis accelerates the progression of HIV disease stages [2]. This coexistence of PTB and HIV infection increased the risk of morbidity and mortality. ART has decreased TB incidence in HIV‐infected patients [3]. The waning of the immune system increased *Mycobacterium tuberculosis* susceptibility and progression of dormant tuberculosis bacilli to endogenous reactivation of latent incidence in the lung [4]. Pulmonary complications have been one of the commonest causes of morbidity and mortality since the advent of the AIDS pandemic and this further led to the parallel pandemic of tuberculosis in some sub-Saharan African populations where 10%–15% suffered from multiple infections [2, 5]. According to the World Health Organization (WHO) report, in 2019, there were an estimated 10.1 million new cases of TB and 1.7 million new deaths, making TB the leading cause of death from a single infectious agent (ranking above HIV/AIDS) [6]. The reactivation of latent pulmonary TB is higher among people living with HIV. Africa is the second TB burden region (25%) after Southeast Asia (44%) [6, 7] and is responsible for one-third of TB/HIV-associated deaths of children living with HIV [8, 9].

Globally, about 36.7 million patients were living with HIV/AIDS and 2.1 million people became newly infected in 2015. Sub-Saharan Africa countries account for the largest proportion, with 25.6 million people living with HIV [1, 6]. Likewise, in 2018, there were about 251,000 deaths from TB among PLWHIV, which accounts for 33% of total deaths associated with HIV, which is much higher than the case fatality rate expected ≤5% by WHO [1, 10].

People living with HIV (PLWHIV) who have active TB disease were prone to several adverse outcomes. In particular, seropositive children have the risk of increased mortality, developing AIDS-defining event, and loss to follow uprate [7]. The Ethiopian Federal HIV and AIDS Prevention and Control Office estimated that the single National HIV/AIDS is among the top ten high burden counties, in which 31% of TB patients are living with HIV [9, 10]. Physiologically, lack of productive cough and sputum for children living with HIV deters early diagnosing and treatment of HIV-associated PTB [11, 12]. Epidemiological data on the magnitude of PTB comorbidity is important to control and prevent both diseases. In this regard, facility-based studies conducted in Ethiopia have indicated that HIV-associated PTB has an increasing trend from 5.0% in Northern Ethiopia [2] to 7.2% in Southwest Ethiopia [5]. Additional studies are required in different regions of Ethiopia to identify high-risk children's cases management. Therefore, this study was intended to estimate magnitude and predictors for the occurrence of pulmonary TB among children who received antiretroviral therapy in 2020.

## 2. Methods

### 2.1. Study Design, Area, and Populations

Metekel zone is one of the three administrative zones found in the Benishangul Gumuz region in Northwest Ethiopia, geographically located within 34° 10′N and 37° 40′E and latitude 09° 17′N and 12° 06′ N. This zone had seven administrative district Woredas with two primary and one-referral hospitals. Gilgel Belles is the capital of Metekel zone, which is located at 565 and 394 km away from Addis Ababa and Assosa, on the way to GERD projects (Ethiopian Great Renaissance Damp at Abay River). Apart from other health service, all these health institutions have been providing ART care services since 2007 [4, 13].

### 2.2. Study Design

**Figure 1:**
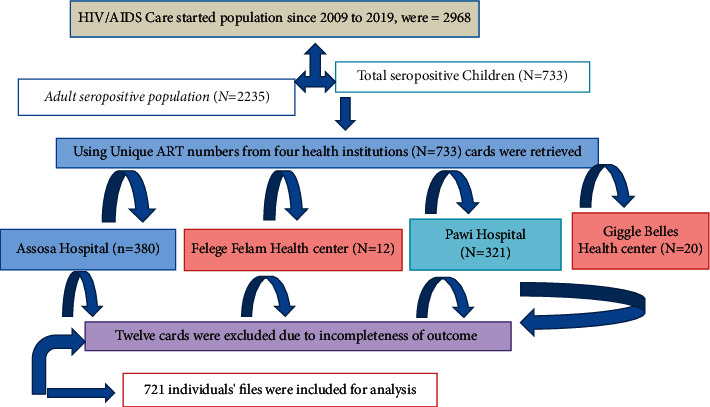
Schematic presentation sampling procedures for the study of seropositive children.

A facility-based historical cohort study design was employed among 721 seropositive children in two hospitals and two health centers from January 1, 2009, to December 31, 2019. All these health facilities provide health care services for an estimated 2968 total catchment populations as depicted in [4, 14] Figure 1.

### 2.3. Study Populations

All recoded pediatrics patients under the age of 15 years who received ART initiated and started care in four selected public health facilities were considered as source population. All HIV-infected children who had at least one month of ART follow-up from January 1, 2009, to December 31, 2019, were included. Patients taking anti-TB treatment at the time of HIV/AIDS enrolment were excluded from the study.

### 2.4. Sample Size Determination and Sampling Techniques

The sample size was calculated by using the formula for survival analysis considering the following parameters, two-sided significance level (*α* = 5%), *Za*/2 = *Z* value at 95% confidence interval = 1.96, power (*Z*_*B*_) = 80%, and AHR = adjusted hazard ration = 1.8 [14].

The final sample size (*n*)=Event/*P*(Event)=(*Z*_*a*/2_+*Z*_*B*_)2/*θ*^2^*p*(1 − *p*)=(*za*/2+*ZB*)/*p*(1 − *p*)(lnHR)2 [15].

(1) $$\begin{array}{c} \theta =\ln\left(\text{HR}\right),\\ \text{HR}=e^{\theta }, \end{array}$$

where alpha (*a*) = 0.05, beta (*β*)  = 0.2, AHR is hazard ratio, *E* is number of events, *N* (sample size) = *E*/*P* (*E*), where *P* (*E*) = probability of event, and *P* = cumulative occurrence of pulmonary TB, 7.2% from this reference [5]. The final sample size was determined as 512.5 after adding 15% contingency for data incompleteness. However, from 1, 2009, to December 31, 2019. Totally, 738 charts were found within the four institutions. Since this data is manageable in resources and could have been assumed to the increased power of inference, we included all charts without sampling procedure.

### 2.5. Outcome Ascertainment

The PTB incidence is considered as an event of interest after HIV/AIDS care clinic started. Children who were lost, died, or transferred out or did not develop the events until the last visit were considered censored, whereas variables including sociodemographic such as age, sex, residence, family size, parental history of TB contact, plus clinical factors like WHO clinical stage, baseline cluster of differentiation (CD4 count), Hgb, functional status, and nutritional status like stunting, wasting, and being underweight were considered as independent variables.

### 2.6. Operational Words

Smear-positive pulmonary tuberculosis: at least one sputum smear examination is positive for AFB on direct-microscopy. Smear-negative pulmonary tuberculosis: sputum specimens negative for AFB and radiographical abnormalities were consistent with active TB and the decision by a clinician to treat with a full course of antituberculosis chemotherapy. Extrapulmonary tuberculosis is clinically consistent with active extra-pulmonary TB and bacteriologically confirmed by AFB of one specimen from an extrapulmonary site for *Mycobacterium tuberculosis* and a decision by a clinician to treat with a full course of anti-TB chemotherapy [1].

### 2.7. Operational Words

SAM DefinitionAccording to the WHO, weight-for-height ≤–3 Z-score, or mid-upper-arm circumference <115 mm, or presence of bilateral edema with failed appetite test should be classified for inpatient care [16]. Undernutrition was defined as a child having one of the description H/Age Z-score < −2 or W/Age Z-score < −2 or W/H Z-score < −2 SD [17, 18]. Moreover, the details description is here below as moderate underweight was defined as children having W/Age Z-score < −2 SD or severe was defined as children; moderate stunting was defined as children having H/Age Z-score < −2 SD. Severe stunting was defined as children having H/Age Z-score < −3 SD [18, 19]. Moderate wasting was defined as children having W/H Z-score < −2 SD. Severe wasting was defined as children having W/H Z-score < −3 SD *n* having W/Age Z-score < −3 *Z* score [17, 18].

A CD4 Count CD4 below the threshold level was classified based on the age of the child's (i.e., infants CD4<1500/mm3, 12–35 months < 750/mm3, 36–59 months < 350/mm3, and ≥5 years < 200/mm3) [10]. ART Adherence for pediatrics is classified based on the percentage of drug dosage calculated from the total monthly doses of ART drugs; Good >95%, fair 85–94%, and poor <85%. Seropositive children: According to FMOH Continuum of HIV services refers to a comprehensive package of HIV prevention, diagnostic, treatment, care, and support services provided for people at risk of HIV infection or living with HIV and their families” children aged <15 years were classified and treated seropositive children [10, 20].

### 2.8. Data Collection Instruments

A standard and pretested data extraction form was used to extract the required information from Ethiopia's Federal Ministry of Health Pediatrics antiretroviral therapy (ART) follow-up [11]. Before the actual data collection, the prepared checklists were pretested on 36 case notes of follow-up in seropositive children from Jawi Hospital. The two-day training was given for six-diploma nurse's data collectors and two-degree public health officers of supervisors on the objective of the study, variables of interest, and maintaining data confidentiality. Strict follow-up and supervision were carried out during data collection by the principal investigators and feedback was given on daily basis. The collected data were checked for inconsistencies, coding errors, completeness, accuracy, and missing values.

### 2.9. Data Collection Procedures and Quality Assurance

To assure the quality of data, data collectors and supervisors were trained about how and what information they should collect from the medical records for one day. The checklist was pretested on 5% of randomly selected charts, which were not included in the actual study. After the pretest, necessary modification of the data collection tool was made. Strict follow-up and supervision were carried out during data collection by principal investigators and feedback was given daily. Individual records with incomplete data during data collection were excluded. The collected data were first being checked and cleaned for completeness.

### 2.10. Statistical Analyses

Data were entered using Epi-Data version 4.2 statistical software and exported to STATA (SE) R-14 version statistical software for further analysis. The WHO AntroPlus Version 1.04 and ENA for Smart Software was used to generate the *Z* score *(WAZ, HAZ, WHZ/BAZ*) to define the nutritional status of seropositive children. We used the Cox regression hazards, regression model, to estimate mortality incidence and predictors during risky successive follow-up. Kaplan–Meier survival analysis was used to determine the cumulative probability of death for all seropositive children and the meantime to death. Variable with *P* value <0.25 in bivariable Cox regression analysis was included in the multivariable Cox regression model. We tested the assumptions of Cox Proportional Hazard Models using Schoenfeld residuals. Variables with an AHR and 95% confidence interval (CI) and a *P* value <0.05 were claimed as significant predictors SAM associated mortality of seropositive children.

## 3. Result

### 3.1. Sociodemographic Characteristics of Seropositive Children

As depicted in Figure 1, data of seven hundred thirty-two (*N* = 732) seropositive children who initiated ART care were reviewed. During the data extraction process, 12 pediatrics files were excluded due to previous TB treatment and incomplete documentation. Based on this, the overall response rate found was 721/732 (99.6%). More than half (384, 53.26%) of participant cases were females in gender, and 510 (70.74%) of them were from urban areas. The largest percentage of participant cases was categorized under the age group 11–15 years, which accounted for 389 (53.96%) of the total subjects. The overall mean (±SD) age of participant children was found to be 118.4 ± 38.24 months (Table 1).

### 3.2. Clinical and Medication Characteristics

From the total 721 participant children, more than one-third (258, 35.78%) of cases had at least one type of opportunistic infection (OI). The most frequent OIs (107 (41.47%) and 100 (38.7%)) were diarrheal disease and bacterial pneumonia, respectively.

Moreover, slightly less than half (293, 40.64%) of cases were on AZT-3TC-NVP of ART regimen. Of the total, 237 (33.1%) and 202 (28.02%) participant children were on WHO stage I & II. More than half (419, 58.2%) of participant cases addressed cotrimoxazole preventive therapy (CPT) (Table 2).

### 3.3. Baseline Nutritional Characteristics

Of the total 721 seropositive children nutritionally, 16.6%, 19.9%, and 21.4% were moderately wasted, underweight, and stunted, respectively. Nearly, one-in-five (124, 17.2%) of cases had an inpatient admission history for severe acute malnutrition (SAM) treatment (Table 3).

### 3.4. Incidence of Pulmonary TB

About 721 participants' children were followed up for 16678.07 person per month risk of observation. During the follow-up period, 8.79% (*n* = 63) of new cases of TB occurred. The majority, 52.4% (*N* = 36/63) vs. 38.2% (*n* = 25/63) cases, were smear negative and smear positive pulmonary TB as documented, respectively. However, the remaining 3.4% (*n* = 2) of cases were extrapulmonary TB (EXPTB). Regarding clinical presentations of SNPTB cases, 27 (72.7%) and 23 (60.6%) had a history of high-grade fever and night sweating, respectively. The overall incidence of PTB in this study was determined as 5.8 per 100 child years (95% CI: 4.58, 7.5). In addition, the incidence of those two new TB cases was estimated as 0.21 per 100 child years risks of observations (Table 4).

### 3.5. Predictors for Incidence of PTB

As shown in Table 5 in the multivariable Cox regression analysis, after adjustment and controlling of certain confounding in the final model, four variables were found significantly associated with PTB incidence. Based on this, the risks of developing PTB for children who were not given CPT were more than two times increased as compared with those who were addressed CPT at baseline (AHR = 2.5: 95% CI, 1.84–4.74, *P* < 0.021). Likewise, the risks of developing PTB infection for seropositive children being nutritionally curved height for age (HFA) ≤−3 *Z* score) or being severely stunted were three times (AHR = 2.9: 95% CI, 1.2–7.8, *P* < 0.03) higher as compared with participant children having normal percentiles (HFA, >−2 *Z* score). Moreover, hemoglobin levels had a high predictive value for incident TB; indeed, baseline hemoglobin ≤10 mg/dl was four times increase the hazard of developing PTB as compared to children having ≥10 mg/dl (AHR = 4.02: 95% CI, 2.1–8.1, *P* < 0.001). Similarly, seropositive children who did not address IPT had significant association with PTB occurrence as compared with their peers who have addressed IPT (AHR = 2.4; 95% CI: 1.2; 5.1, *P*=0.001) (Table 5).

## 4. Discussion

At the end of the cohort of 721 HIV-infected children, 90 children (12.4%) died, 24 (3.3%) were lost to follow-up, 68 (15.1%) were transferred to other health institutions, and 539 (74.7%) remained in the follow-up. Moreover, the present study revealed that the crude incidence of TB among children living with HIV/AIDS was determined as 8.7% (6.8; 11.04). This report is higher than the finding from Debre Tabor hospital 5.0% [2], yet it is comparable with finding stated in Arba Minch (7.2%) [5] and Tanzania (8.5%) [21] hospitals. This might be due to similarity in the study setting and the association of protective effects of ART. In the contrary, this report is lower than the finding from Zimbabwe (12.01%) [22] and Yaoundé, Cameroon (61.8%) [23]. The possible elucidation for the difference may be the size of the study population and the higher burden of tuberculosis in resource-limited settings [1]. In addition, HIV waned the immune system and accelerated viral replication for the depletion of CD4 count [10], which precipitated the new episode of lethal opportunistic infections [24]. This can be reduced by early addressing of CPT and IPT, which is inexpensive and highly effective for reducing loads of endogenous reactivation of latent PTB [25]. Equivalent to this report, however, were seropositive children who missed CPT and had significantly associated with risks of developing PTB. This is consistent with a previous document in Northwest Ethiopia and Gondar hospitals [14, 26]. Moreover, in line with research finding with Northwest Ethiopia, Zaire, and Cameroon [4, 23, 27], our study indicated seropositive children who did not take IPT at baseline had a risk of developing PTB. Consistent with research findings in Northwest Ethiopia [4], concomitant administration of IPT with ART after undertaking in-depth active TB signs, power to have demoted more than ninety-six percent of new TB incidence for children living with HIV. Irrationally, nearly two in five of (37.45%) participant children did not take IPT in our study.

Furthermore, result in our research revealed that seropositive children who missed CPT were significantly associated with risks of developing PTB. This discovery is comparable with the previous description in Gondar [28] and Adam hospitals [12]. Cotrimoxazole prophylaxis is safe, inexpensive, and highly effective in reducing morbidity and mortality among HIV-infected infants and children. The World Health Organization (WHO) recommends that all HIV-exposed infants be started on cotrimoxazole prophylaxis at four to six weeks of age, to provide adequate prevention against early opportunistic infections (OIs). This is particularly critical for HIV-infected infants. Data from a randomized clinical trial conducted in Zambia demonstrated the effectiveness of cotrimoxazole prophylaxis in reducing mortality and morbidity among HIV-infected children, despite high levels of bacterial resistance [6, 10, 14]. One of the main challenges countries must address to scale up cotrimoxazole prophylaxis is the absence of mechanisms to systematically identify and follow up HIV-exposed infants at and after birth. If HIV-infected children are to be identified early in the course of their disease—a prerequisite for receiving cotrimoxazole prophylaxis and early ART—systems need to be in place to ensure that health workers consider the possibility of HIV infection in infants at birth and at all clinics or other health encounters thereafter [1, 4, 10, 29]. Our finding also indicated that the median (±IQR) time of TB reported and the overall TB incidence (IDR) were determined as 17.8 (±11) months and 5.86 per 100 child years (95% CI: 4.58, 7.5), respectively. The human immune deficiency virus (HIV) is the strongest risk factor for endogenous reactivation of pulmonary tuberculosis (PTB) through target reduction of CD4 and T-lymphocytes, with rapid bacilli reactivation after children are already exposed to infections [14].

Our study also indicated that participant children at baseline being nutritionally severely stunted were independently associated with PTB occurrence as compared with those who have WHO curve ≥−2 *Z* score. This is similar to finding s in Adama [30], Tanzania [31], and Uganda and Zimbabwe hospitals [32]. This might be due to HIV infection increasing nutrient mal-absorption due to metabolic alterations that culminate in weight loss and stunting with time leading to early exposure for opportunistic infections [11]. Comparably, the existence of rapid viral replication consumed body energy and creates an arena for developing deadly opportunistic infections [33, 34]. This finding also showed that children having hemoglobin ≤10 mg/dl were independently related to HIV-associated PTB occurrence as compared with HIV-infected countergroups. This is in line with the study finding in Adama hospital [30], Gondar hospital [12], Northern Ethiopia [28], Dar es Salaam, Tanzania [31], and England [35]. This is due to hemoglobin levels having a high predictive value for tuberculosis incidence [36].

### 4.1. Limitation of the Study

The retrospective nature of this study is one of the limitations of this study. Due to this, some of the clinically important predictor variables that have been independently associated with the incidence of TB occurrence in other studies, like the educational status of children and economic status of the family, were not included in these studies.

## 5. Conclusion

We found a high burden of pulmonary TB among children living with HIV/AIDS as compared with previous reports in Ethiopia. Cases at baseline not taking IPT and CPT, being severely stunted, and having low hemoglobin (≤10 mg/dl) levels were found to be at higher risk to PTB occurrence. Besides, intensified screening for provisions of isoniazid preventive therapy to children living with HIV/AIDS is highly recommended.

## Figures and Tables

**Table 1:** Baseline sociodemographic characteristic of HIV positive children who received ART in selected public health facility, Northwest Ethiopia, 2020 (*N* = 721).

Variables	Categories	Frequency	Percent
Sex	Male	337	46.7
	Female	384	53.3
Age of children	≥5 years	78	10.8
	6–10	254	35.2
	11–15 years	389	53.9
HIV disclosure status of children	Disclosed	158	21.9
	Not disclosed	563	78.1
Age of caregivers	≤45 years	244	38.8
	<45 years	477	66.1
Resident	Urban	510	70.8
	Rural	211	29.3
Marital status of the caregiver	Single	115	15.9
	Married	498	69.1
	Divorced	82	11.4
	Widowed	26	3.6
Family size of caregivers	≤2	227	31.4
	3–5	462	64.1
	≥6	32	4.4
HIV status of caregivers	Positive	550	76.3
	Negative	91	12.6
	Unknown	80	11.1
Religions of caregivers	Orthodox	381	52.8
	Muslim	152	21.2
	Protestant	139	19.3
	Catholics	49	6.8
Occupational status of caregivers	Farmer	99	13.7
	Merchant	337	46.7
	Employer	124	17.2
	Laborer worker	161	22.3
Parental status of care children	Both alive	381	52.8
	Paternal orphan	135	18.2
	Maternal orphan	108	14.9
	Both orphaned	97	13.5

**Table 2:** Clinical and hematologic characteristics of seropositive children attending ART care in selected public health facilities (*N* = 721), 2020.

Variables	Categories	Number	Frequency
Dietary counseling during follow-up	Yes	465	64.5
	No	256	35.5
Admission history of SAM	Yes	124	17.3
	No	597	82.6
Opportunistic infection (OI at baseline)	Yes	258	35.8
	No	453	64.2
Types of ART regimen	D4t-3TC-NVP	48	6.6
	D4t-3TC-EFV	26	3.6
	AZT-3TC-NVP	293	40.6
	AZT-3TC-EFV	165	22.8
	TDF-3TC-EFV	104	14.4
	AZT-3TC-LPV/R	36	4.9
	ABC-3TC-NVP	25	3.5
	ABC-3TC-EFV	24	3.3
ART regimen change	Yes	211	29.3
	No	510	70.7
Functional status (age ≤5 years)	Appropriate	69	71.9
	Delay	15	15.6
	Regression	12	12.5
Developmental history (age >5 years)	Working	488	77.9
	Ambulatory	87	13.9
	Bedridden	51	8.15
Adherence	Good	356	49.4
	Faire	177	24.5
	Poor	188	26.1
WHO clinical stage	I	237	32.8
	II	202	28.1
	III	170	23.6
	IV	112	15.5
Isoniazid preventive therapy (IPT)	Yes	451	62.5
	No	270	37.4
Cotrimoxazole preventive therapy	Yes	419	58.1
	No	302	41.9
CD4 count per mm^3^	Below the threshold	308	42.7
	Above threshold	413	57.3
Hemoglobin level	≤10 g/dl	229	31.7
	>10 g/dl	492	68.2
Types of opportunistic other than TB	Bacterial pneumonia	79	30.6
	Diarrhea	74	28.8
	Meningitis	9	3.6
	PCP	6	2.33
	Skin dermatitis	7	2.7
	Kaposi's sarcoma	5	1.9
	Acute/chronic otitis media	9	3.5
	Others	3	1.18
Duration on ART	≤36 months	223	30.93
	36–72 months	295	40.92
	73–180 months	203	28.16
Current status of children	On follow-up	539	74.76
	Transferred into other health institution	68	9.43
	Lost to follow-up	24	3.3%
	Died	90	12.48
Maternal PMTC follow-up history	Yes	487	67.6
	No	234	32.4
MUAC	≤11.5 cm	270	37.45
	>11.5 cm	451	62.55
Children status during data collection	Died	87	12.07
	Survived	634	87.93
Incidence of PTB/TB during follow-up	Event	63	8.7%
	Censored	658	91.2%

**Table 3:** Nutritional status of seropositive children attending ART cares in selected health facility at Benishangul Gumuz regions, 2020 (*n* = 721).

Variables			Frequency
Weight-for-age (WFA)	Normal growth curve	WAZ ≥ −2 *Z* score	491 (68.1
	Moderate underweight	WAZ −2-3 *Z* score	144 (19.9%)
	Severe underweight	WAZ ≤ −3 *Z* score	86 (11.9%)
Height-for-age (HFA)	Normal growth	HAZ ≥ −2 *Z* score	428 (59.4%)
	Moderate stunting	HAZ −2-3 *Z* score	154 (21.4%)
	Severe stunting	HAZ ≤ − 3 *Z* score	139 (19.2%)
Weight-for-height (WFH)	Normal growth curve	WHZ ≥ −2 *Z* score	531 (73.5%)
	Moderate wasting	WHZ −2-3 *Z* score	119 (16.6%)
	Severe wasting	WHZ ≤ −3 *Z* score	71 (9.9%)
SAM admission history by sex, age, and residence (*N* = 124)	Sex	Male	65/124 (52.4%)
		Female	59124 (47.6%)
	Resident	Rural	70/124 (56.45%)
		Urban	54/124 (43.5%)
	Age	≤60 months	33/124 (26.6%)
		61–120 months	42/124 (33.8%)
		121–179 months	49/124 (39.5%)

**Table 4:** Clinical presentations of seropositive children before confirmed PTB/TB diagnosed in selected public facility Northwest Ethiopia, 2020.

Types of TB (*N* = 63)	Frequency (%)	Presentation	Cough (%)	Fever (%)	Night sweating (%)	Weight loss (%)	Hemoptysis (%)
SNPTB	36 (57.2)	Present	31 (88.5%)	27 (72.7)	23 (60.6%)	23 (60.6%)	8 (15.5%)
		Absent	5 (15.5%)	9 (27.7%)	13 (39.3%)	13 (39.3%)	24 (72.3%)
SPPTB	25 (41.2)	Present	18 (70.8%)	24 (95.5%)	15 (58.3%)	14 (54.2%)	7 (25%)
		Absent	7 (29.2%)	1 (5.5%)	10 (42.6%)	11 (45.8%)	18 (75%)
EXPTB	2 (3.5%)	Present	1 (50%)	2 (100)	1 (50%)	2 (100)	0
		Absent	1 (50%)	0	1 (50%)	0	2 (100)

SNPTP: smear negative pulmonary TB; SPPTB: smear positive pulmonary TB; EXPTB: extrapulmonary TB.

**Table 5:** Bivariable and multivariable Cox-proportional hazard analysis for predictors of PTB among children who received ART in a selected public health facility in Northwest Ethiopia (*N* = 721).

Variables	Categories	CHR (95% CI)	AHR	*P* value
Sex	Male	1.8 (1.1, 3.0)	1.5 (0.83;2.7)	0.11
	Female	1	1	
Age	≤5 years	1	1	
	6-10 years	1.3 (0.85, 1.7)	1.7 (0.8; 4.2)	0.27
	11–15 years	1.9 (1.2, 3.8)	1.5 (0.64;3.5)	0.34
Residence	Rural	3.4 (2.5, 9.7)	1.7 (0.76; 3.8)	0.19
	Urban	1	1	
Family size	≤2	1	1	
	3–5	2.5 (1.2; 4.5)	2.1 (0.9; 13.7)	0.05
	≥6	1.3 (0.4; 4.8)	2.3 (0.94; 9.2)	0.31
Disclosure status	Yes	2.5 (2.2; 7.5)	1.2 (0.6; 2.2)	0.42
	No	1	1	
IP	Given	1	1	
	Not given	5.6 (2.1, 8.3)	2.4 (1.2; 5.1)^*∗*^	0.01^*∗*^
CPT	Given	1	1	
	Not given	3.7 (2.8; 7.8)	2.5 (1.4–4.7)^*∗*^	0.02^*∗*^
ART drug adherence	Good	1	1	
	Fair	2.1 (1.7;4.1)	1.8 (0.97–2.4)	0.31
	Poor	3.1 (2.05; 6.3)	1.2 (0.68–2.2)	0.11
Height-for-age (HFA)	HFA ≥ −2 *Z* score	1	1	
	HFA −2-3 *Z* score	2.1 (1.5; 2.8)	1.2 (0.8–2.7)	0.17
	HFA ≤ −3 *Z* score	3.9 (3.8; 5.1)	2.9 (1.2–7.8)^*∗*^	0.03^*∗*^
WHO clinical stages	Stage I & II	1	1	
	Stage III & IV	5.1 (4.9; 6.4)	1.8 (0.8–2.5)	0.12
Levels of hemoglobin	> 10 mg/dl	1	1	
	≤10 mg/dl	9.6 (5.13; 18.0)	4.(2.1; 8.1)	0.01^*∗*^
Baseline MUAC	≤11.5 cm	2.4 (1.5; 3.9)	1.7 (0.89; 2.5)	0.11
	>11.5 cm	1	1	
Weight-for-height (WFH)	WAZ ≤ −3 *Z* score	2.8 (1.5; 3.3)	1.7 (0.6; 4.59)	0.26
	WAZ −2-3 *Z* score	2.6 (1.92; 2.8)	1.61 (0.91; 2.85)	0.10
	WAZ ≥ −2 *Z* score	1	1	
Times of follow-up	≤36 months	1	1	
	36–72 months	1.9 (0.76; 4.8)	0.86 (0.29; 2.6)	0.80
	73–180 months	2.4 (1.99; 8.53)	1.5 (0.7; 3.02)	0.36
SAM admission history (SAM)	Yes	2.4 (1.9; 6.3)	1.3 (0.68; 2.5)	0.69
	No	1	1	
CD4/mm^3^ count	Below threshold	2.2 (1.7; 4.1)	1.4 (0.82; 5.1)^*∗*^	0.21
	Above threshold	1	1	

## Data Availability

The data sets of this research are available from the main author on reasonable request.

## Abbreviations

TB: Tuberculosis
CPT: Cotrimoxazole preventive therapy
Hgb: Hemoglobin
IPT: Isoniazid preventive therapy
AHR: Adjusted hazard ratio
CHR: Crude hazard ratio
FMOH: Ethiopian Federal Ministry of Health
MUAC: Midupper arm circumference
PLWHIV: People living with HIV
WFH: Weight-for-height
WFA: Weight-for-age
HFA: Height-for-age
SAM: Severe acute malnutrition
WFH: Weight-for-height
SD: Standard deviation
IQR: Interquartile range.
